# Renal Damage Frequency in Patients with Solitary Kidney and Factors That Affect Progression

**DOI:** 10.1155/2015/876907

**Published:** 2015-12-10

**Authors:** T. Basturk, Y. Koc, Z. Ucar, T. Sakaci, E. Ahbap, E. Kara, F. Bayraktar, M. Sevinc, T. Sahutoglu, A. Kayalar, A. Sinangil, C. Akgol, A. Unsal

**Affiliations:** Department of Nephrology, Sisli Etfal Research and Educational Hospital, 34371 Istanbul, Turkey

## Abstract

*Background*. The aim of this study is to assess renal damage incidence in patients with solitary kidney and to detect factors associated with progression.* Methods*. Medical records of 75 patients with solitary kidney were investigated retrospectively and divided into two groups: unilateral nephrectomy (group 1) and unilateral renal agenesis/dysplasia (group 2). According to the presence of kidney damage, each group was divided into two subgroups: group 1a/b and group 2a/b.* Results*. Patients in group 1 were older than those in group 2 (*p* = 0.001). 34 patients who comprise group 1a had smaller kidney size (*p* = 0.002) and higher uric acid levels (*p* = 0.028) than those in group 1b at presentation. Uric acid levels at first and last visit were associated with renal damage progression (*p* = 0.004, 0.019). 18 patients who comprise group 2a were compared with those in group 2b in terms of presence of DM (*p* = 0.038), HT (*p* = 0.003), baseline proteinuria (*p* = 0.014), and uric acid (*p* = 0.032) levels and group 2a showed higher rates for each. Progression was more common in patients with DM (*p* = 0.039), HT (*p* = 0.003), higher initial and final visit proteinuria (*p* = 0.014, for both), and higher baseline uric acid levels (*p* = 0.047).* Conclusions*. The majority of patients with solitary kidney showed renal damage at presentation. Increased uric acid level is a risk factor for renal damage and progression. For early diagnosis of renal damage and reducing the risk of progression, patients should be referred to a nephrologist as early as possible.

## 1. Background

The solitary kidney can be caused by three main reasons: being born with a single kidney, losing one because of a disease, or an injury or donating a kidney to a family member or a friend who has lost one of their own. The condition of a person who was born with a single kidney is known as renal agenesis. This occurs in about one of every 750 births. Being born with a single kidney is more common in males, and the left kidney is absent more often than the right one. Renal dysplasia is another congenital defect which refers to the condition of being born with an unfunctional kidney [1, 2].

There is increasing evidence suggesting that either inherited or acquired nephron loss is associated with the increased risk of proteinuria and renal insufficiency. Experimental models of renal ablation have demonstrated the important role of hyperfiltration at the onset of renal impairment [3, 4].

Patients with unilateral nephrectomy maintain the remaining kidney function over time, as it has been described in healthy kidney donors. This is a selected population, which has extensively ruled out the presence of renal disease prior to nephrectomy. Among the population of solitary kidney related to other causes, such as tumors, tuberculosis, or stones, there is possibility of potential bilateral involvement. Some authors have considered that the measurement of clearance as 75% of the normal value should be set as normal [5]. We proposed this study to assess renal damage incidence in patients with SK and to detect factors associated with the progression.

## 2. Patients and Methods

We performed a retrospective analysis of patients with solitary kidney in our outpatient clinic in the period between January 2012 and May 2013. Medical records of 75 patients with solitary kidney were collected and the patients were divided into two groups: unilateral nephrectomy (group 1) and unilateral renal agenesis/dysplasia (group 2). According to the presence of renal damage, each group was divided into two subgroups as group 1a/b and group 2a/b.

The diagnosis of unilateral renal agenesis/dysplasia was established to the patients, radiologic evaluations of whom (excretory urography, renal ultrasonography, computed tomography, and renal scans) discovered a solitary kidney without any history of surgical removal of the contralateral kidney. Patients with the history of kidney donation and partial nephrectomy were excluded.

As our patients refer to our outpatient clinic in every one to 3 months in our clinical practice, data which represent regular follow-up every three months were obtained from our clinical records. The duration of follow-up between the initial evaluation and the last visit, death, or onset of chronic dialysis was recorded in every case. Age, gender, presence of diabetes mellitus (DM) and hypertension (HT), medical history, causes of nephrectomy, duration between the nephrectomy and first outpatient clinic visit, duration of time until the recognition of agenesis/displasia, systolic and diastolic blood pressure, body mass index (BMI) measurements, levels of serum creatinine, BUN, uric acid, Glomerular Filtration Rate (GFR) (24 hours' urine collection and* MDRD* formula), microalbuminuria, proteinuria, and urine sediment examination were recorded at presentation and during follow-up. Also, if they had received pharmacological treatment with angiotensin converting enzyme inhibitors (ACEI) or antagonists of the angiotensin type II (ARB), these treatments were noted.

Blood pressure values which were measured with a standard mercury sphygmomanometer at every visit were obtained from the records. Systolic and diastolic blood pressures were measured after 5 minutes of rest in a sitting position; the average of 2 measurements was recorded. BMI was calculated as the weight in kilograms divided by the square of the height in meters. Renal damage was defined as a creatinine higher than 1.4 mg/dL in men and 1.3 mg/dL in women or CCr lower than 60 mL/min or proteinuria ≥300 mg/day. Progression was considered when Scr increased above 50% of its baseline level. Other major variables that we have considered were the duration of time till the persistence of proteinuria (at least in 3 consecutive measurements) which is higher than 0.3 g/24 hr, time to requirement of renal replacement therapy, and changes in proteinuria.

Statistical analysis was performed using the SPSS 17.0 software package (SPSS Inc., Chicago, IL, USA). Kolmogorov-Smirnov tests were used to test the normality of data distribution. The data were expressed as arithmetic means and standard deviations. The Chi-squared test was used to compare the categorical variables between the groups. Independent sample *t*-tests and Mann-Whitney *U* tests were used between groups for normally and abnormally distributed continuous variables, respectively. Spearman and Pearson tests were used in correlation analysis. A two-sided *p* value 0.05 was considered to be statistically significant.

## 3. Results

Seventy-five patients were evaluated in our study; 52% (*n* = 39) of these patients were male and 48% (*n* = 36) were female, with a mean age of 51.6 ± 12.6. The solitary kidneys of 44 patients were evaluated as group 1 (26 males, mean age of 57.9 ± 13 years) and those of 31 patients as group 2 (13 males, mean age of 44.2 ± 13.1 years). All of group 1 patients underwent radical nephrectomy of one kidney. The causes of nephrectomy were inactive lesions of bilateral renal tuberculosis in 4 patients, bilateral nephrolithiasis with superimposed pyelonephritis in 5 patients, and bilateral chronic pyelonephritis (history of recurrent urinary tract infections and acute pyelonephritis) in 6 patients; 2 patients had undergone nephrectomy because of an accident and 14 patients had solitary kidney following the nephrectomy for any renal cancer. The main characteristics of the study groups are shown in Table 1. Patients of group 1 were older than group 2 (*p* = 0.001) and there were no significant differences between groups statistically (*p* > 0.05) (Table 1).

At the time of diagnosis, renal damage was present in a total of 53. A higher prevalence of renal damage was seen among patients (79%) in group I than patients in group II (58%). Higher creatinine/decreased creatinine clearance was the most frequently described renal damage as it has been seen in 44 patients. The distribution of study groups is shown in Table 2.

In group 1, 9 patients showed normal renal function at presentation, whereas the remaining 35 cases, some of whom presented with renal insufficiency as well, had proteinuria. Group 1a had smaller longitudinal renal size (*p* = 0.002) and higher uric acid levels (*p* = 0.028) than group 1b (Table 2). During the follow-up period, 9 patients from group 1 remained normal, whereas the remaining 1 developed proteinuria and two patients required hemodialysis.

The univariate risk factors analysis for progression of renal damage showed statistical significant differences between groups 1a and 1b in terms of initial and eventual uric acid levels (*p* = 0.004, 0.019 and *r* = 0.485, 0.365, resp.).

In group 2, 13 patients showed normal renal function at presentation, whereas the remaining 18 cases, some of whom presented with renal insufficiency, had proteinuria. Patients of group 2a were compared with those of group 2b according to the presence of DM (*p* = 0.038), HT (*p* = 0.003), baseline proteinuria (*p* = 0.014), and uric acid (*p* = 0.032) levels and these variables were found higher in group 2a (Table 3). 13 patients from group 2 remained normal, whereas the remaining 1 developed proteinuria and 2 patients started hemodialysis. The univariate risk factors analysis for progression of renal damage showed statistically significant differences between groups 2a and 2b in presence of DM and HT (*p* = 0.039, 0.003 and *r* = 0.373, 0.392, resp.), higher initial and last visit proteinuria levels (*p* = 0.014, 0.014 and *r* = 0.459, 0.460, resp.), and higher baseline uric acid (*p* = 0.047 and *r* = 0.385) (Table 4).

In our study, 72% of the whole patients were recorded as hypertensive. Patients with solitary kidney in each group showed no differences in terms of the hypertension frequency (80.5%, group 1 and 67.7%, group 2). 63% of hypertensive patients were on treatment of ACE or ARB. During the follow up period, number of the patients with renal damage did not increase significantly, and only 4 patients required hemodialysis (Table 5).

## 4. Discussion

Unilateral nephrectomy has been the most frequent cause of solitary kidney in the studied sample. It is likely that among adult patients neoplasms outweigh pathologies. Most of renal agenesis/dysplasia patients represent an incidental finding at some abdominal imaging test. In the majority of our patients with solitary kidney, renal damage was detected at the time of the presentation, especially in patients with unilateral nephrectomy. Increased uric acid level is a risk factor of renal damage occurrence and its progression in two groups. In patients with renal agenesis/dysplasia, the presence of DM and HT and proteinuria comprise a higher risk for progression of renal damage.

Adaptive phenomenon occurs at the solitary kidney as a result of the diminished number of nephrons. Increases in both size and functional capacity of the kidney have been shown in animal and human studies [6, 7].

Increased renal blood flow and glomerular pressures may cause a mechanical stimulation for renal growth [8, 9]. According to Delanaye et al., [10] the adaptive capacity of the nephrons is lower in obese and elderly patients. The research carried on animal and human kidneys shows that, in the course of aging, there is a progressive loss of renal parenchyma so that one-third to one-half of renal nephrons are lost by old age [11].

Aging is associated with structural and functional changes in the kidney [12]. The rate of GFR decline with age varies widely among individuals. Usually beginning after 30–40 years of age, GFR progressively declines at an average rate of 8 mL/min/1.73 m² per decade. The rate of decline may accelerate after the age of 50–60 years [13]. Structurally, there is progressive loss of renal mass with age, particularly in the cortex, leading to a decreased number of glomeruli and an increase in the proportion of sclerotic glomeruli. A decline in GFR has been observed in both cross-sectional and longitudinal studies [14].

In fact, the studies on renal functional reserve in patients with acquired solitary kidney have often shown a partially or completely blunted response in several years following nephrectomy [15, 16]. Thus, acquired solitary kidney may possibly be more vulnerable to additional stress than congenital forms of solitary kidney. Wang et al. [17] showed that the patients with congenital solitary kidney with a kidney length less than 120 mm or proteinuria had a higher risk of renal insufficiency.

In our study, renal sizes of patients in both groups increased similarly, the age at diagnosis for nephrectomy is higher in patients with nephrectomy. We also found higher prevalence of renal damage in nephrectomized patients. Additionally, in patients with nephrectomy who show renal damage, kidney sizes were smaller than the patients with congenital solitary kidneys. We have thought that it is because of the adaptive capacity of the nephrons decreased by age and it could also be related to the progressive decrease of GFR by age.

In humans, a 50% decrease in renal mass causes an increase in GFR within hours and, in 30 days, generally stabilizes at 75–85% of the predonation values, and serum creatinine increases by approximately 20% [7]. In 27 patients who were monitored for more than 20 years, Dousdampanis et al. [18] found an immediate increase in creatinine clearance of about 34%, peaking at 2–6 months postoperatively and then reaching a plateau [15]. The time interval from nephrectomy to GFR decrease varies and depends on several factors. Twenty-five to 30% of patients undergoing unilateral nephrectomy in childhood (mean age of 7 years) developed proteinuria and renal insufficiency after a median follow-up of 25 years. The infant with a GFR less than 50% of normal for age at the time of diagnosis is even more likely to experience progressive renal insufficiency [19]. The study demonstrated that 20% to 50% of the study young adults with a congenital solitary functioning kidney (SFK) required dialysis by the age of 30 years, leading to the advice to monitor all patients with an SFK from childhood [20].

We suggest that information about the age at presentation of renal injury and about clinical factors that differentiate the situation, with or without renal injury, would guide the timing and frequency of clinical follow-up.

The observations of Baudoin et al. [6] and Wikstad et al. [21] noticed that renal functions tend to decrease slowly but significantly with increasing follow-up time since childhood in adults with SK [6–17, 19–21]. In the same manner, an inverse relationship between GFR and follow-up time was found only in acquired solitary kidney (ASK) but not in congenital solitary kidney (CSK) [22]. González et al. [23] performed a retrospective study of 54 patients with severe reduction in renal mass (33 patients with URA and 21 with RK) and showed that 63% of patients had renal damage. Follow-up duration was 100 ± 72 months.

In our study, renal damage was found in 71% of all patients at presentation. High rate of renal damage and the average age of a similar but shorter follow-up period were determined. There is lack of adequate data to guide us in the management of patients with SK whom we might identify as at risk of progressive renal damage because of the reduced number of nephrons. The SK represents a particular entity in nephrology, requiring special attention from specialists.

In group 1, the duration between the nephrectomy and the reference to our outpatient clinic for the first time was 6 years and patients were under follow-up for 43.05 ± 35.33 months. While it was not known whether renal damage was present before nephrectomy or not, renal damage was found in 82% of these patients at presentation. In group 2, the first reference to our outpatient clinic was 77.2 ± 63.8 years after the first diagnosis of renal agenesis/displasia; the follow-up duration was 42.65 ± 31.43 and renal damage was found in 68% of these patients.

The follow-up durations and renal damage rates were not different significantly both between two groups and in total.

The time interval from nephrectomy to decline in glomerular filtration rate varies and depends on several factors. An increase in serum uric acid level occurs during the CKD and aggravates the deterioration of kidney function. Uric acid may contribute to kidney fibrosis mainly by inducing inflammation, endothelial dysfunction, oxidative stress, and activation of the renin-angiotensin system. Besides, hyperuricemia induces alterations in renal hemodynamics via afferent arteriolopathy and contributes to the onset and progression of kidney fibrosis [24].

Obermayr et al. [25] reported that patients with uric acid level greater than 7 mg/dL were at increased risk for CKD, including a 1.74-fold risk in men and 3.12-fold risk in women. Hyperuricemia and increased fractional urate excretion have recently been reported in patients with SK, and it is thought that these factors might be harmful to the structure and function of the remaining kidney [18]. Jeon et al. found that increased preoperative uric acid was an independent predictor of a low preoperative glomerular filtration rate and new onset chronic kidney disease in patients with renal cell carcinoma who underwent nephrectomy [26].

Our analyses revealed that the uric acid levels were found high in both patients with renal agenesis and nephrectomized patients with renal insufficiency. On the other hand, uric acid and decreased GFR independently correlated in patients with SK.

Hypertension and proteinuria are also consistently demonstrated to be independent risk factors for the progression of renal damage. Evidence shows that renal mass (i.e., quantity of nephrons) is a major risk factor for the development of hypertension and glomerular injury. Blood pressure increases as one ages, and renal function decreases [27]. In studies based on clinical assessment of blood pressure, the prevalence of hypertension was variable ranging from 0% to 50% [28, 29].

In 22 patients studied by Rugiu et al. (9 patients with solitary kidney due to renal agenesia and 13 due to nephrectomy), the frequency of arterial hypertension was significantly higher in the group of patients with renal agenesia than in the nephrectomy group [30]. Dursun et al. recorded a systemic hypertension in up to 26% of their patients by means of 24 h ABPM (32% with acquired solitary kidney and 22% with unilateral renal agenesis [31]). In our study, hypertension, proteinuria, or renal failure was present in approximately two-third of patients with renal agenesis/dysplasia. Those with HT and proteinuria had a higher risk of progression to renal insufficiency.

The SK identifies patients with increased microalbuminuria or proteinuria and perhaps early treatment with group ACEI or ARB appears to be effective in proteinuria reduced renal progression rates, fibrogenesis, and protection of renal aging [32]. In our study, hypertension was recorded in 72% of the whole study group. 63% of hypertensive patients were on treatment of ACE-i or ARB. Our study did not give the clear beneficial influence of ACEI or ARB treatment on the outcome of patients showing with/without renal damage at presentation. Follow-up time for renal functions remained stable in both groups and only 5.3% of patients received HD treatment.

## 5. Conclusions

Prevalence of renal damage was found higher among patients with solitary kidney, especially in patients with unilateral nephrectomy. Increased uric acid levels were related to renal damage formation and progression. Patients who had hypertension and proteinuria held a higher risk for progression to renal insufficiency. RAS blockers did not provide beneficial effects on reducing progression rates to renal insufficiency. The majority of our patients with solitary kidney showed renal damage at the time of presentation. For early diagnosis of renal damage and reducing the risk of progression, patients should be referred to a nephrologist at early stages.

## Figures and Tables

**Table 1:** Clinical characteristics at presentation.

	Group 1 (*n*: 44)	Group 2 (*n*: 31)	*p*
Age (years)	57.98 ± 13.02	**44.23 ± 13.13**	0.001
Gender M/F	26; 18	**13; 18**	NS
BMI kg/m^2^	27.22 ± 4.76	25.50 ± 4.80	NS
Kidney size cm	119.06 ± 12.51	119.11 ± 17.43	NS
Duration of follow-up (months)	43.05 ± 35.33	42.65 ± 31.43	NS
At the time of diagnosis (months)	119.35 ± 110.06	77.2 ± 63.8	0.024
SBP mmHg	135.61 ± 17.04	130.32 ± 24.83	NS
DBP mmHg	80.24 ± 12.35	80.94 ± 12.72	NS
GFR (initial) mL/min/1.73 m^2^	51.50 ± 29.22	*61.19 ± 28.92*	NS
GFR (last) mL/min/1.73 m^2^	48.55 ± 27.21	60.45 ± 34.10	NS
Creatinine (initial) mg/dL	1.74 ± 1.04	1.57 ± 1.05	NS
Creatinine (last) mg/dL	2.03 ± 1.77	1.83 ± 1.52	NS
Proteinuria (initial) g/24 h	0.52 ± 0.75	1.01 ± 1.57	NS
Proteinuria (last) g/24 h	0.61 ± 1.02	0.873 ± 1.30	NS
Uric acid (initial) mg/dL	6.67 ± 1.9	6.27 ± 1.9	NS
Uric acid (last) mg/dL	6.5 ± 1.6	5.7 ± 1.4	0.028
Diabetes mellitus	7 (17.1%)	5 (16.1%)	NS
Hypertension	33 (80.5%)	21 (67.7%)	NS
RAS blockade	21 (63.6%)	16 (64%)	NS

**Table 2:** Solitary kidney-associated renal damage rate.

	Total 75		Group 2 (*n*: 31)		Group 1 (*n*: 44)	
	Initial	Last	Initial	Last	Initial	Last
With renal damage	53 (71%)	42 (56%)	18 (58%)	17 (55%)	35 (79%)	35 (80%)
Proteinuria	26 (35%)	28 (37%)	11 (35%)	12 (39%)	15 (34%)	16 (36%)
Renal failure	44 (59%)	46 (61%)	14 (45%)	14 (45%)	30 (68%)	32 (73%)
Renal failure and proteinuria	17 (23%)	22 (29%)	7 (22%)	9 (29%)	10 (23%)	13 (29%)
Without renal damage	22 (29%)	23 (31%)	13 (42%)	14 (45%)	9 (20%)	9 (20%)

**Table 3:** Clinical features of nephrectomy patients at presentation.

	Group 1a (*n*: 34) CKD (+)	Group 1b (*n*: 10) CKD (−)	*p*
Age (years)	59.38 ± 13.84	53.2 ± 8.7	NS
Gender M/F	20/14	6/4	NS
BMI kg/m^2^	27.8 ± 4.6	22	NS
Kidney size cm	116.07 ± 11.19	132.5 ± 9.23	0.002
Duration of follow-up (months)	41.24 ± 35.75	49.2 ± 34.98	NS
At the time of diagnosis (months)	116.59 ± 117.02	129.78 ± 83.43	NS
SBP mmHg	135.94 ± 17.75	134.44 ± 15.09	NS
DBP mmHg	80.63 ± 12.43	78.89 ± 12.69	NS
GFR (initial) mL/min/1.73 m^2^	43.65 ± 26.62	78.20 ± 21.36	NS
GFR (last) mL/min/1.73 m^2^	39.09 ± 20.21	80.7 ± 23.56	NS
Proteinuria (initial) g/24 h	0.64 ± 0.82	0.13 ± 0.09	NS
Proteinuria (last) g/24 h	0.74 ± 1.13	0.15 ± 0.14	NS
Creatinine (initial) mg/dL	1.94 ± 1.1	1.03 ± 0.24	NS
Creatinine (last) mg/dL	2.33 ± 1.91	1.02 ± 0.26	NS
Uric acid (initial) mg/dL	7.1 ± 1.7	4.9 ± 1.5	0.028
Uric acid (last) mg/dL	6.9 ± 1.5	5.5 ± 1.3	NS
Diabetes mellitus	5 (14.7%)	2 (20%)	NS
Hypertension	26 (76.5%)	7 (70%)	NS
RAS blockade	15 (44.1%)	6 (60%)	NS

**Table 4:** Clinical features of unilateral renal agenesis/dysplasia patients at presentation.

	Group 2a (*n*: 18) CKD (+)	Group 2b (*n*: 13) CKD (−)	*p*
Age (years)	47.22 ± 13.09	40.08 ± 12.47	NS
Gender M/F	7/11	6/7	NS
BMI kg/m^2^	27 ± 4.583	21	NS
Kidney size cm	115.81 ± 20.40	123.91 ± 11.08	NS
Duration of follow-up (months)	51.33 ± 35.56	30.62 ± 20.18	NS
SBP mmHg	133.33 ± 26.78	126.15 ± 22.18	NS
DBP mmHg	82.78 ± 15.26	78.38 ± 7.89	NS
GFR (initial) mL/min/1.73 m^2^	44.72 ± 21.39	84 ± 21.72	NS
GFR (last) mL/min/1.73 m^2^	42.17 ± 29.32	85.77 ± 22.21	NS
Proteinuria (initial) g/24 h	1.53 ± 1.75	0.056 ± 0.06	0.014
Proteinuria (last) g/24 h	1.30 ± 1.44	0.089 ± 0.10	0.014
Creatinine (initial) mg/dL	2.01 ± 1.19	0.94 ± 0.16	NS
Creatinine (last) mg/dL	2.49 ± 1.72	0.93 ± 0.18	NS
Uric acid (initial) mg/dL	6.8 ± 2.1	5.3 ± 1.1	0.032
Uric acid (last) mg/dL	6.0 ± 1.6	5.2 ± 1.1	NS
Diabetes mellitus	5 (27.8%)	0	0.038
Hypertension	15 (83.3%)	6 (46.2%)	0.003
RAS blockade	10 (55.5%)	6 (46.2%)	NS

**Table 5:** Clinical features of two groups of patients treated with/without RAS.

	Group 1 (*n*: 33)		Group 2 (*n*: 21)	
	RAS (−) (*n*: 12)	RAS (−) (*n*: 8)	RAS (−) (*n*: 8)	RAS (+) (*n*: 21)
Initial GFR (mL/min/1.73 m^2^)	34.6 ± 24.6	45.2 ± 27.7	45.2 ± 27.7	60.1 ± 28
Last GFR (mL/min/1.73 m^2^)	32.8 ± 31.2	44.5 ± 33.4	44.5 ± 33.4	55 ± 21.1
Initial proteinuria (g/24 h)	1.1 ± 1.25	1.3 ± 1.92	1.3 ± 1.92	0.37 ± 0.56
Last proteinuria (g/24 h)	1.10 ± 1.62	0.95 ± 0.98	0.95 ± 0.98	0.48 ± 0.79
